# First Case Report of Primary Carnitine Deficiency Manifested as Intellectual Disability and Autism Spectrum Disorder

**DOI:** 10.3390/brainsci9060137

**Published:** 2019-06-13

**Authors:** José Guevara-Campos, Lucía González-Guevara, José Guevara-González, Omar Cauli

**Affiliations:** 1 “Felipe Guevara Rojas” Hospital, Pediatrics Service, University of Oriente, El Tigre-Anzoátegui 6034, Venezuela; joguevara90@hotmail.com; 2 “Felipe Guevara Rojas” Hospital, Epilepsy and Encephalography Unit, El Tigre-Anzoátegui 6034, Venezuela; draluciagonzalez@gmail.com; 3 “Miguel Pérez Carreño” Hospital, Pediatrics Service, Caracas 1020, Venezuela; joose.guevara@gmail.com; 4 Department of Nursing, University of Valencia, 46010 Valencia, Spain

**Keywords:** carnitine, autism, intellectual disability, muscle, rare disease

## Abstract

Systemic primary carnitine deficiency (PCD) is a genetic disorder caused by decreased or absent organic cation transporter type 2 (OCTN2) carnitine transporter activity, resulting in low serum carnitine levels and decreased carnitine accumulation inside cells. In early life, PCD is usually diagnosed as a metabolic decompensation, presenting as hypoketotic hypoglycemia, Reye syndrome, or sudden infant death; in childhood, PCD presents with skeletal or cardiac myopathy. However, the clinical presentation of PCD characterized by autism spectrum disorder (ASD) with intellectual disability (ID) has seldom been reported in the literature. In this report, we describe the clinical features of a seven-year-old girl diagnosed with PCD who presented atypical features of the disease, including a developmental delay involving language skills, concentration, and attention span, as well as autistic features and brain alterations apparent in magnetic resonance imaging. We aim to highlight the difficulties related to the diagnostic and therapeutic approaches used to diagnose such patients. The case reported here presented typical signs of PCD, including frequent episodes of hypoglycemia, generalized muscle weakness, decreased muscle mass, and physical growth deficits. A molecular genetic study confirmed the definitive diagnosis of the disease (c.1345T>G (p.Y449D)) in gene *SLC22A5*, located in exon 8. PCD can be accompanied by less common clinical signs, which may delay its diagnosis because the resulting global clinical picture can closely resemble other metabolic disorders. In this case, the patient was prescribed a carnitine-enriched diet, as well as oral carnitine at a dose of 100 mg/kg/day. PCD has a better prognosis if it is diagnosed and treated early; however, a high level of clinical suspicion is required for its timely and accurate diagnosis.

## 1. Introduction

Systemic primary carnitine deficiency (PCD; Online Mendelian Inheritance in Man (OMIM): 212140) is an autosomal recessive disorder, associated with decreased carnitine uptake across plasma membranes because of a deficiency in organic cation transporter type 2 (OCTN2), encoded by the *SLC22A5* gene on chromosome 5q31 [1,2]. Carnitine deficiency causes defective fatty acid oxidation and utilization for energy production. When fatty acids cannot be used, glucose is consumed without regeneration via gluconeogenesis, resulting in hypoglycemia [3,4,5]. The disorder has a frequency of about 1:40,000 to 1:120,000 newborns, and its incidence varies depending on ethnicity. It is highly prevalent in individuals with ancestry originating in the Faroe Islands, because 5% of this population are heterozygotes or carriers of the mutated gene [6,7]. 

Primary carnitine deficiency classically presents as a hypoketotic, hypoglycemic encephalopathy, which is often associated with hepatomegaly, elevated transaminase levels, hyperammonemia, cardiomyopathy, pericardial effusion, muscle weakness, altered gastrointestinal motility, and recurrent attacks of abdominal pain and diarrhea, along with anemia and repeated infections during early life [8,9,10,11]. Secondary carnitine deficiency occurs in other metabolic disorders, including organic acidemia and fatty acid oxidation defects, such as very-long-chain acyl-CoA dehydrogenase, medium-chain acyl-CoA dehydrogenase, long-chain hydroxyacyl-CoA dehydrogenase, or carnitine palmitoyltransferase II deficiencies. Drugs such as cyclosporine and valproate can also cause carnitine deficiency, renal tubular dysfunction, and malnutrition, and complete parenteral nutrition may also result in secondary carnitine deficiency [11]. 

Metabolic disorders accompanied by altered carnitine biosynthesis have recently been linked to neurodevelopmental disorders, such as autism spectrum disorder (ASD), in which the deletion of the trimethyllysine hydroxylase gene, a key gene in carnitine biosynthesis, has been associated with non-dysmorphic autism in males [12,13]. In addition, biochemical alterations leading to secondary carnitine deficiency have been reported in a subgroup of patients with ASD [14,15,16,17]. To date, no reports have linked PCD caused by organic OCTN2 deficiency with ASD.

Here we report the interesting and novel case of a female child in which we identified a PCD gene mutation. This patient presented atypical signs for PCD, such as ASD and ID, as well as brain magnetic resonance imaging (MRI) alterations. We hope the description of this phenotype–genotype will help guide clinicians in their differential diagnosis of children presenting ASD and developmental growth delay. 

## 2. Case Report

This was a seven-year-old schoolgirl who, starting from three years of age, began presenting frequent episodes of sweating, vomiting, fatigue, generalized muscle weakness, and low glucose levels. These episodes became more recurrent, with shorter intervals between their presentation. The girl also presented growth and language delay, involving language skills, concentration, and attention span, as well as ASD and mild ID, so she was referred to a pediatric neurologist. 

The patient was born by caesarean section at 36 weeks because of premature placental detachment, and had an Apgar score of 8. The girl was the product of a third pregnancy, resulting from a non-consanguineous relationship, and there was no family history of metabolic or neurological disease. Her birth weight was 2.360 kg (25th percentile) and length was 45 cm (50th percentile), and she received normal immunizations. 

At the time of her physical examination by the pediatric neurologist, she had the following characteristics: weight 15 kg (<10th percentile), height 107 cm (<10th percentile), head circumference 50 cm (50th percentile), and blood pressure 107/60 mmHg; fever was absent. Her general condition was regular, no dysmorphic features or skin blemishes were observed, and cardiopulmonary auscultation was normal. Neurological examination revealed normal cranial nerves, as well as isochoric and normoreactive pupils. She presented mild hypotonia, reduced muscular strength in both her arms and legs (grade IV/V), and decreased osteotendinous reflexes; her superficial and deep sensitivity were normal. 

The patient had achieved head support at 12 months, a stable seated position for the first time at 24 months, and she had started to autonomously walk at 30 months. Her first raw words had appeared at 12 months, but she was still unable to produce complex sentences at the time of the examination. The patient’s language skills were poor for her age, she had a moderate intellectual disability (ID), and she was inattentive and impulsive. The girl had poor eye contact and displayed stereotyped and repetitive movements; according to the Peabody Developmental Motor Scales (PDMS-2 scale) and (Battelle Developmental Inventory (BDI-2), she exhibited a global developmental delay. The final diagnosis was moderate ID and a pervasive developmental disorder not otherwise specified (according to the Diagnostic and statistical manual of mental disorders (DSM-V) criteria and the “Autism Diagnostic Interview—Revised” [18]).

**Figure 1:**
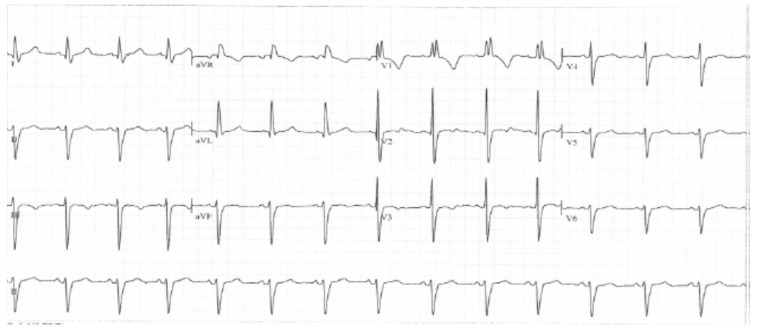
Electrocardiogram of the patient, which shows normal findings for a child of her age.

Her electrocardiogram (Figure 1), echocardiogram, chest radiograph, electroencephalogram, thorax radiography, electromyography, and conduction velocity analyses of the peroneal and tibial nerves were all normal.

**Figure 2:**
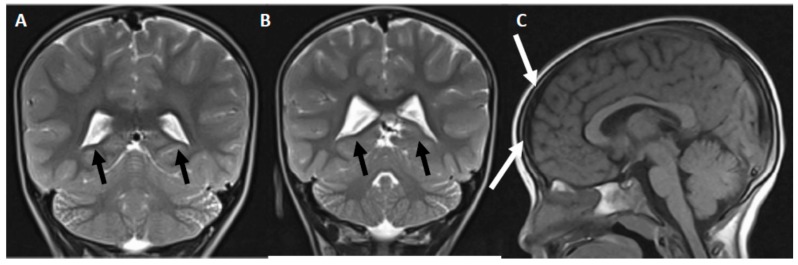
Brain structural alterations observed in the patient. (**A**,**B**) Axial, magnetic resonance, T2-weighted images showing discrete asymmetry of the lateral ventricles. Note that the right side is larger (indicated by the black arrows). (**C**) Sagittal, magnetic resonance, T1-weighted image showing discrete frontal subdural hygroma (indicated with white arrows).

Brain MRIs showed discrete asymmetry of the lateral ventricles (the right side was larger) and discrete bifrontal subdural hygroma (Figure 2). 

## 3. Blood Laboratory Tests

Laboratory tests showed the following blood plasma results: hemoglobin: 12.1 g/dL; hematocrit: 34%; leukocytes: 7.7 × 10^−9^/L; 58% neutrophils; 38% lymphocytes; 3% monocytes; 1% eosinophils; platelets: 294 × 10^−9^/L; glucose: 61 mg/dL; urea: 24 mg/dL; creatinine: 0.61 mg/dL; alkaline phosphatase: 258 U/L; calcium: 9 mg/dL; aspartate aminotransferase: 8 U/L; alanine aminotransferase: 16 U/L; total bilirubin: 0.4 mg/dL; direct bilirubin: 0.2 mg/dL; indirect bilirubin: 0.15 mg/dL; cholesterol: 195 mg/dL; ammonia: 24 μM ; creatine kinase-muscle/brain: 3 U/L; creatine kinase: 52 U/L; thyroxine: 40 ng/dL; triiodothyronine: 2.6 ng/mL; and thyroid-stimulating hormone: 1.25 μU/mL. 

Analysis of her blood gases showed the following: pH: 7.48; pCO_2_: 25.8 mmHg; pO_2_: 70.6 mmHg; HCO_3_: 21.4 mEq/L; base excess: −3.1 mEq/L; and anion gap: 18.5 mEq/L. For the serum electrolytes, the following were found: sodium: 141 mEq/L; potassium: 4.90 mEq/L; and chlorine: 101 mEq/L. 

Regarding the fasting and postprandial parameters, we found the following: fasting lactate: 2.6 mM; postprandial lactate: 3.5 mM; fasting pyruvate: 0.08 Mm; postprandial pyruvate: 0.12 mM; fasting lactate pyruvate ratio: 32.5; postprandial lactate pyruvate ratio: 29.2; and postprandial glycemia: 78 mg/dL. 

The concentration of organic acids in the urine, blood amino acids, biotinidase, and cystine/homocysteine were all within the normal range. Levels of free carnitine in the plasma were 4.3 µM (normal range: 25–50 µM). The blood plasma acylcarnitine panel results were normal, except for 3-OH-iso-butyrylcarnitine, which was 0.13 µM (normal range is <0.5 µM).

## 4. Genetic Analysis

We performed a molecular genetic analysis of a panel of 22 fatty acid oxidation-disorder nuclear genes. The coding regions were amplified using a capture-based method, and sequenced using the Illumina platform. This sequencing method is based on reversible dye terminators that allow single bases to be identified as they are introduced into DNA strands (performed in Baylor Miraca Genetics Laboratories, Houston, TX, United States). The molecular analysis showed a heterozygous pathogenic variant, c.1345T>G (p.Y449D, p.Tyr449Asp) in the *SLC22A5* gene located in exon 8 (Figure S1). This mutation is a missense variant that has been reported in patients with primary carnitine deficiency [5].

## 5. Treatment and Patient Evolution

The girl was started on a treatment regime of 200 mg/kg/day of L-carnitine (which was well-tolerated), in addition to a vitamin B complex (50 mg each of vitamins B1 and B2, 15 mg of B3, 2 mg of B6, and 10 mg of B12), divided into two daily doses, because she did not eat fruits. This patient is currently nine years old and attends a special school, which she has accepted well. Her muscle weakness and language skills have improved, but she remains inattentive. She still has autistic features, although her ID has improved by 15 IQ points relative to her cognitive function before the PCD diagnosis and carnitine treatment (increasing to the 65th IQ percentile from the 50th, a year before treatment started). Even with treatment, she has still presented some episodes of hypoglycemia (<60 mg/dL).

## 6. Discussion

Here we presented a clinical case of childhood-onset PCD with both rare clinical characteristics (autistic features, ID, and developmental delay) and common ones (episodes of hypoglycemia and proximal muscle weakness). Classic PCD presents with dilated cardiomyopathy and elevated creatine kinase, or with clinical signs of infantile metabolic involvement, such as episodes of hypoketotic hypoglycemia, hyperammonemia, hepatomegaly, elevated transaminase levels, and hepatic encephalopathy (poor feeding, irritability, and lethargy) [5,11,19]. We hope that this description is useful to neurologists and pediatricians, and may give them more reason to suspect a diagnosis of PCD, even when the patient phenotype does not fully fit the classical presentation of the disease. 

Our molecular analysis showed that the patient carries a heterozygous missense variant, c.1345T>G (p.Y449D, p.Tyr449Asp), in the *SLC22A5* gene located in exon 8, and expression studies of variants showed that Y449D has a functional effect [5,20]. PCD is caused by both homozygous or compound heterozygous mutations in *SLC22A5* on chromosome 5q31, and new cases of heterozygous mutations have recently been described [21,22]. ASD has only been reported in cases of PCD in this study, and in one previous case report of a child with developmental delay [23]; therefore, ASD does not appear to occur with an increased frequency among patients with PCD. 

Perhaps this apparent discrepancy can be explained by the fact that PCD diagnoses in patients with systemic signs (e.g., hypoglycemia or muscle weakness) are usually made when the patient is aged two years or less. This means that some infants may be put on carnitine supplementation early enough to prevent brain carnitine deficiency and its resulting functional alterations in the brain. Likely more important is the fact that the brain can synthesize carnitine, and that the carnitine deficiency in PCD probably has a stronger effect on tissues outside the brain. 

In the case reported here, PCD was diagnosed at a later stage, and because of this delay, more time passed without carnitine supplementation—likely leading to poorer brain development and a more severe phenotype. In line with this interpretation, this patient’s response to carnitine treatment was mild to moderate, and the ASD features were not reversed. However, we cannot exclude the possibility that other genetic or epigenetic alterations related to carnitine metabolism and transport or other nutrients may have contributed to the phenotype of this patient. 

This clinical case supports the hypothesis that *SLC22A5* heterozygous deficiency might be a risk factor for autism, and thus warrants future studies, because ASD may be preventable in this subgroup [24]. Confirming this hypothesis, the Faroe Islands population, which has a high proportion of heterozygous or homozygous *SLC22A5* deficiency, also has an increased prevalence of ASD. However, this prevalence is not as high as would be expected based on the prevalence of the *SLC22A5* mutation, probably because of the consumption of a diet containing red meat (high in carnitine) from early ages, which may have helped prevent some cases of ASD in this population [24].

In line with the clinical characteristics of our patient, heterozygous mutations in the carnitine transporter gene *SLC22A5* are not associated with cardiomyopathy. However, benign cardiac hypertrophy can develop in adulthood, and so our patient will require close monitoring for this potential complication [20]. Pioneering studies on the relationship between genotype and phenotype in PCD have demonstrated that nonsense mutations are more likely to be associated with reduced carnitine transport, and are more prevalent in symptomatic individuals, while erroneous mutations allow some carnitine transport activity, and are more common in asymptomatic individuals [23,25]. However, patients with the same mutation can have different clinical manifestations [23,26], and these dissimilarities are likely because of interactions between other genetic alterations and non-genetic factors [2,27]. 

Other than our case, developmental delay and ID associated with PCD has only been reported once before [23]. Therefore, we suggest that testing for plasma carnitine levels might be considered in the differential diagnosis of child developmental delays. Thus, PCD should also be included in the framework of possible genetic causes of developmental delay and IDs. Importantly, the timely diagnosis of these diseases can be used to tailor suitable therapeutic treatments for these patients. Our report extends previously published findings that correlated carnitine deficiency to a subgroup of patients with ASD with a trimethyllysine hydroxylase gene deletion. This is a key gene in carnitine biosynthesis [12,13], as well as in a subgroup of ASD patients with secondary carnitine deficiency and mitochondrial metabolism impairment [14,15,16,17,24,28]. Furthermore, our report supports the hypothesis that carnitine deficiency is a risk factor for ASD [24,29].

Newborns are not routinely screened for PCD in Venezuela, meaning that diagnoses are often missed or delayed. The presence of low levels of carnitine and short acylcarnitines in blood plasma is a clue that an underlying metabolic disorder associated with primary or secondary carnitine deficiency should be suspected. However, it is important to note that the transport of maternal carnitine through the placenta can lead to false negatives in newborns and exclusively breastfed infants in the first days of life, as was the case in our patient. 

Mitochondrial *β*-oxidation of fatty acids is an essential energy-producing pathway during times of increased metabolic demand. Carnitine plays an important role in the transfer of long-chain fatty acids into the mitochondria for *β*-oxidation, especially in tissues like the liver, skeletal muscles, and cardiac muscles [11,19,30]. The case we present here met the criteria for energy impairment, as shown by the increases in the lactate/pyruvate ratio. Alterations in the plasma concentrations of lactate, pyruvate, and related ratios are not specific to any one disorder, and so the results must be interpreted in the context of each individual’s clinical presentation and other laboratory results [19,31,32]. 

Abolished or reduced carnitine transporter activity impairs the proper use of fatty acids as an energy source during periods of fasting or stress. In line with this, some of the episodes of hypoglycemia experienced by our patient occurred during seasonal and common respiratory infections. This could result in acute metabolic decompensation, and so hypoglycemia and the frequency of food intake should be strictly controlled in these patients [4,11].

Very few studies have performed brain MRIs in patients with PCD; however the brains of infants with PCD show symmetric white matter abnormalities in the bilateral frontal cortex, caudate nuclei, and genus of the internal capsule, leaving the temporal–occipital lobes relatively unaffected [4,33,34,35,36]. T2-weighted hyperintensity and decreased diffusion at the level of corona radiata, cerebral and cerebellar white matter, cerebellar peduncles, and corticospinal and corticobulbar brainstem tracts have been reported in severe cases of PCD with hypoglycemic hypoketotic encephalopathy [34]. In contrast, in our patient, the MRIs showed unspecific brain alterations: discrete lateral ventricle asymmetry and bifrontal subdural hygroma.

The latter is an accumulation of cerebrospinal fluid, usually without blood, localized under the dural meningeal space [37], and is usually derived from chronic subdural hematomas. Bifrontal subdural hygromas are most common in elderly people after minor traumas and in children after severe infections [38]. In most cases, in the absence of any acute trauma or severe neurological symptoms, the presence of a small subdural hygroma on these scans is considered incidental, and when asymptomatic (as in our case), they do not require any treatment [39].

According to international guidelines, patients with PCD must receive carnitine administration and an adequate dietary intake of carnitine (mainly from meat) [11]. Theoretically, carnitine administration should start before irreversible organ damage occurs. Although we observed a low to moderate clinical improvement in our patient, the lack of full reversal of the symptoms after two years of carnitine treatment might be secondary to permanent brain structure or brain circuit alterations. 

## Supplementary Materials

The following are available online at https://www.mdpi.com/2076-3425/9/6/137/s1, Figure S1: Identification of c.1345T>G (p.Y449D, p.Tyr449Asp) mutation in the *SLC22A5* gene.
