# Supplementary material for: First Case Report of Primary Carnitine Deficiency Manifested as Intellectual Disability and Autism Spectrum Disorder
Source: Brain Sci. 2019 Jun 13;9(6):137. doi: 10.3390/brainsci9060137 (PMC6628273; doi:10.3390/brainsci9060137)
Supplement: Supplementary file 1 [file brainsci-09-00137-s001.pdf]

Supplementary Material: First Case Report of Primary Carnitine Deficiency Manifested as Intellectual Disability and Autism Spectrum Disorder

José Guevara-Campos [1], Lucía González-Guevara [2], José Guevara-González [3], and Omar Cauli [4,*]

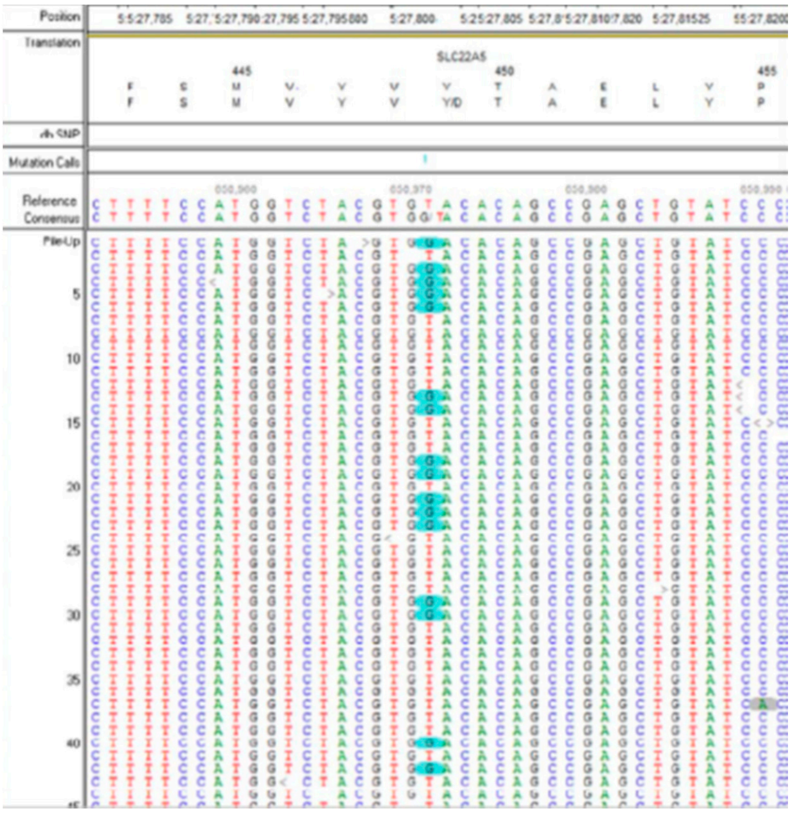

Figure S1. Identification of c.1345T>G (p.Y449D, p.Tyr449Asp) mutation in the *SLC22A5* gene.
